# Cardiac Insulin Resistance and MicroRNA Modulators

**DOI:** 10.1155/2012/654904

**Published:** 2011-07-31

**Authors:** Lakshmi Pulakat, Annayya R. Aroor, Rukhsana Gul, James R. Sowers

**Affiliations:** ^1^Department of Internal Medicine, School of Medicine, University of Missouri, Columbia, MO 65212, USA; ^2^Department of Nutrition and Exercise Physiology, School of Medicine, University of Missouri, Columbia, MO 65212, USA; ^3^Diabetes and Cardiovascular Laboratory, School of Medicine, University of Missouri, Columbia, MO 65212, USA; ^4^Harry S. Truman Memorial Veterans' Hospital, Columbia, MO 65201, USA; ^5^Department of Medical Pharmacology and Physiology, School of Medicine, University of Missouri, One Hospital Drive, Columbia, MO 65212, USA

## Abstract

Cardiac insulin resistance is a metabolic and functional disorder that is often associated with obesity and/or the cardiorenal metabolic syndrome (CRS), and this disorder may be accentuated by chronic alcohol consumption. In conditions of over-nutrition, increased insulin (INS) and angiotensin II (Ang II) activate mammalian target for rapamycin (mTOR)/p70 S6 kinase (S6K1) signaling, whereas chronic alcohol consumption inhibits mTOR/S6K1 activation in cardiac tissue. Although excessive activation of mTOR/S6K1 induces cardiac INS resistance via serine phosphorylation of INS receptor substrates (IRS-1/2), it also renders cardioprotection via increased Ang II receptor 2 (AT2R) upregulation and adaptive hypertrophy. In the INS-resistant and hyperinsulinemic Zucker obese (ZO) rat, a rodent model for CRS, activation of mTOR/S6K1signaling in cardiac tissue is regulated by protective feed-back mechanisms involving mTOR*↔*AT2R signaling loop and profile changes of microRNA that target S6K1. Such regulation may play a role in attenuating progressive heart failure. Conversely, alcohol-mediated inhibition of mTOR/S6K1, down-regulation of INS receptor and growth-inhibitory mir-200 family, and upregulation of mir-212 that promotes fetal gene program may exacerbate CRS-related cardiomyopathy.

## 1. Introduction

The confluence of a constellation of interactive maladaptive factors such as hypertension, insulin (INS) resistance, metabolic dyslipidemia, obesity, microalbuminuria, and/or reduced renal function constitute the cardiorenal metabolic syndrome (CRS) [1–6]. Clustering the increasing numbers of these risk factors within an individual heightens metabolic perturbations which, in turn, promote development of cardiovascular diseases (CVD) and type 2 diabetes (T2DM) [5, 6]. The CRS affects more than one-third of the US population and is rising to pandemic proportions worldwide. Overnutrition caused by excessive consumption of diets rich in carbohydrates, largely from highly processed foods and sugar-sweetened beverages (Western diet), positively correlates with the rise of CRS risk factors and CVD [7–14]. Overnutrition results in chronic exposure of cardiovascular (CV) tissues to circulating nutrients, glucose, and INS. All of these factors promote attenuation of INS metabolic signaling and INS resistance independently, and therefore, collectively they exert significant stress on CV tissues [15–19]. Moreover, overnutrition induces activation of the renin-angiotensin system (RAS) which can elevate systemic and tissue angiotensin II (Ang II), a potent vasoconstrictive and proinflammatory hormone. Ang II-mediated activation of Ang II type 1 receptor (AT1R) promotes both INS resistance and CVD, and inhibition of Ang II generation, as well as blocking of AT1R signaling, has proven to be beneficial in treatment of INS resistance [20–32]. Overnutrition also alters adipocyte functions by reducing secretion of anti-inflammatory, anti-ischemic adiponectin and increasing secretion of proinflammatory, prothrombotic adipokines such as resistin [33–35]. Thus, in conditions of overnutrition, cardiac insult is exerted by a plethora of extracellular signals (increased INS and Ang II, an adverse adipokine profile, and excessive glucose, amino acids and lipids) and hemodynamic/neuroendocrine stresses originating from hypertension, hypertrophy, and fibrosis. 

In the initial stages of overnutrition related cardiac INS resistance, several compensatory mechanisms are activated in heart tissue to protect the functions of this vital organ by promoting adaptive compensatory signaling and remodeling. In this context, activation of the nutrient sensor kinase mammalian target for rapamycin (mTOR) in cardiac tissue, under conditions of overnutrition, is particularly noteworthy [36]. The mTOR Complex 1 (mTORC1) serves as a converging point for signals from nutrients, INS and Ang II, and is frequently activated in CV tissues in conditions of overnutrition and aging [36–44]. On the one hand, increased mTORC1-mediated signaling is implicated in left ventricular (LV) remodeling, myocardial infarction, hypertrophic cardiomyopathy, and atherosclerosis [45–48]. Conversely, mTOR is required for exercise-induced adaptive hypertrophy and remodeling [49]. Attenuation of mTORC1 signaling via cardiac specific ablation of Raptor, the scaffolding protein required for association of mTOR with its substrates p70 s6 kinase (S6K1) and eIF4E-binding protein (4E-BP), induces impairment of adaptive hypertrophy and causes heart failure in mice [50–54]. Given the fact that mTOR signaling is required for adaptive cardiac hypertrophy, it is conceivable that activation of mTORC1 in cardiac tissue in response to excess nutrients, Ang II, and INS can be a compensatory mechanism to help the heart cope with overnutrition-related stresses such as hemodynamic overload. In this paper, we describe regulation of overnutrition-related cardiac mTORC1 signaling by inherent protective feedback mechanisms that involve mTORC1-mediated activation of Ang II type 2 receptor (AT2R) and changes in microRNA profiles that, in turn, can potentially downregulate S6K1 expression. 

Chronic alcohol consumption, in the context of overnutrition, is an additionally significant risk factor that promotes cardiac pathology and dysfunction [55]. Alcoholism may be the most common form of drug abuse. Moderate alcohol consumption has been associated with a reduced risk of CVD and improvement of INS resistance [56, 57]. However, chronic alcoholism (excessive and prolonged alcohol consumption with >80 g of ethanol a day for >10 years) can result in alcoholic cardiomyopathy [55–60]. Chronic alcohol treatment inhibits protein synthesis in cardiac muscles and cause protein loss [58–60]. This alcohol effect is mediated by an inhibition of stimulatory phosphorylation of mTOR and S6K1 in cardiac tissue and subsequent downregulation of protein synthesis [58]. We posit that alcohol-mediated inhibition of mTOR/S6K1 activation may contribute to attenuation of an important compensatory mechanism (mTORC1 signaling) that can promote adaptive hypertrophy under conditions of overnutrition. Moreover, chronic alcohol administration is shown to alter microRNA profiles in other tissues. A close examination of these alcohol-regulated microRNAs indicates that some of these microRNAs are also expressed in cardiac tissue. In this paper we explore the possible cardiac outcomes related to impaired myocardial INS metabolic signaling that can occur when alcohol-induced microRNA modulations are superimposed on overnutrition-induced adaptive compensatory signaling mechanisms in heart tissue. 

## 2. Overnutrition-Induced Activation of mTORC1 and mTOR*↔*AT2R Signaling Loop in Cardiac Tissue and Cardiomyocytes

TOR is a 289-kDa serine/threonine protein kinase that is evolutionarily conserved from yeast to man (mTOR) and a member of the phosphatidylinositol 3-kinase- (PI3-K-) related kinase (PIKK) family. Signals that activate the canonical PI3-K-protein kinase B (Akt) pathway (growth factor receptors, Ang II, INS-mediated activation of IRS-1/PI3-K-Akt pathway) result in mTOR stimulation. Akt activates mTOR by directly phosphorylating mTOR at Ser^2448^ [37–42]. Akt also promotes mTORC1 complex formation indirectly since it phosphorylates proline-rich Akt substrate of 40 kDa (PRAS40), the negative regulator of Raptor, and promotes dissociation of PRAS40 from mTORC1 so that Raptor is free to associate with mTOR substrates S6K1 and 4E-BP. Additionally, phosphorylation of tuberous sclerosis complex 2 (TSC2) by Akt results in inhibition of its GTPase activity and thus promotes GTP loading on Rheb (Ras homolog enriched in brain) and Rheb-mediated mTORC1 activation [61]. Amino acids also activate mTORC1 via heterodimeric Rag GTPases that promote translocation of mTORC1 to a membrane-bound compartment that contains the mTORC1 activator, Rheb [62]. Moreover, these diverse mechanisms for activation of mTORC1 by amino acids and INS act in a cooperative manner and provide a physiological explanation for increased mTORC1 signaling in response to overnutrition-related increases in nutrients and INS [63]. However, mTORC1 activation contributes to heart and skeletal muscle INS resistance since mTOR substrate S6K1 is a serine (Ser)/threonine kinase that phosphorylates IRS-1, a critical INS signaling/docking molecule, on specific Ser residues. Excessive Ser phosphorylation of IRS-1 attenuates IRS-1 tyrosine phosphorylation and IRS-1-PI3-K association and subsequent INS metabolic signaling. In this context, mTORC1 activation can induce INS resistance in cardiac tissue. However, it is also conceivable that development of INS resistance in conditions of overnutrition and hyperinsulinemia is actually a compensatory mechanism that may serve to protect cardiac cells from excessive signaling generated by excess INS and nutrients. 

Cardiac mTORC1 activation may play a significant role in increased protein synthesis required for adaptive hypertrophy. In this context, activation of mTORC1 leads to increased translation and cell growth by two mechanisms: first, phosphorylation of Thr^389^ of S6K1 by mTOR results in activation of S6K1 and subsequent phosphorylation of five evolutionarily conserved residues (Ser235, Ser236, Ser240, Ser244, and Ser247) of ribosomal protein S6 (RPS6) that activates RPS6. RPS6 increases translation of 5′TOPmRNAs and protein synthesis. Second, mTOR phosphorylates 4E-BP on Thr^37^ and Thr^46^ and relieves 4E-BP-mediated repression of translation initiation factor eIF4E and thus enhances translation [39, 42, 50, 64–66]. It should be noted that cardiac overexpression of mTOR protects against cardiac dysfunction following LV pressure overload. Conversely, ablation of cardiac raptor results in impairment of adaptive cardiac hypertrophy and causes heart failure in mice [54]. Interestingly, mTOR also protects heart from pathological hypertrophy associated with inflammatory response [52, 54]. These observations imply a delicately balanced mTORC1 function that helps the heart cope with stress induced by exercise, pressure overload, and inflammation. Our recent findings support the notion that in the setting of overnutrition-induced impairment of cardiac INS metabolic signaling, mTORC1 signaling is carefully controlled by protective feedback mechanisms. 

**Figure 1:**
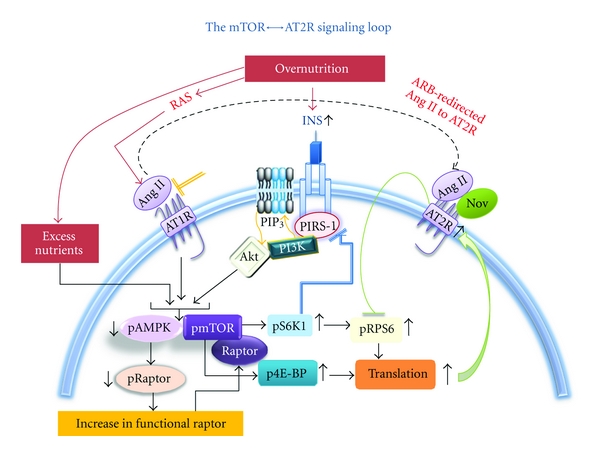
The mTORC1-mediated increase in AT2R expression can lead to AT2R-mediated inhibition of mTOR substrates and modulate mTORC1 signaling.

The CRS rodent model Zucker Obese (ZO) rat is polyphagic due to a mutation in the leptin receptor and serves as a model of overnutrition-induced INS resistance and diastolic heart failure with preserved ejection fraction [67–69]. Importantly, this genetic model manifests biochemical and functional cardiac abnormalities that are seen in obese humans; however, the ZO rat does not progress to overt diabetes until late in life. We observed activation of mTORC1 in the left ventricle of ZO rats; however, a concomitant increase in growth-inhibitory Ang II receptor AT2R was also observed [2]. It is conceivable that the hyperinsulinemic status of ZO rat may simultaneously promote mTORC1 activation since INS activates mTORC1 and increases AT2R expression [38, 70–72]. What is paradoxical is that accumulating evidence indicates that AT2R activation is cardioprotective [73–77]. AT2R inhibits cell growth and mediates the beneficial effects of AT1R blockade and PPAR-*γ* activation, reduces fibroblast growth and myocardial hypertrophy, and mediates the antihypertrophic and antifibrotic effects of AT1R blockade [76–80]. Since AT2R activates phosphatases, it is conceivable that AT2R expression in cardiac tissue in response to overnutrition/hyperinsulinemia can regulate mTORC1 kinase and down-stream signaling. Such regulation could play a significant role in maintaining the delicate balance of mTORC1 activation that promotes compensatory adaptive cardiac hypertrophy to cope with increased hemodynamic load associated with obesity. Our observation that Ang II- and INS-mediated activation of mTORC1 signaling in mouse cardiomyocytes is, in part, responsible for increased AT2R expression (that may, in turn, regulate mTORC1 signaling) supports this notion. Rapamycin treatment elevates glucose intolerance in obese sand rat. We observed that rapamycin-induced inhibition of mTORC1 and siRNA-mediated inhibition of S6K1 attenuated elevation of AT2R expression. Thus, treatments that ablate mTORC1 and inhibit AT2R do not seem to be cardioprotective in overnutrition conditions. Conversely, AT2R agonism in ZO rats by a nine-day infusion (200 *μ*g·kg^−1^·day^−1^) of Novokinin (Nov), an AT2R agonist, reduced the increase in mTORC1 signaling and yet improved myocardial performance. In brief, mTORC1 activation leads to the formation of an mTOR*↔*AT2R signaling loop that can serve as a protective feedback mechanism to balance enhanced mTORC1 signaling in cardiac tissue in conditions of overnutrition-induced INS resistance and RAS activation (Figure 1). 

## 3. Regulation of S6K1 by microRNA in Cardiac Tissue

The microRNAs (miRNA) have emerged as an important group of translational regulators that target and regulate 60% of the mammalian genome [81–84]. Recent studies have shown that cardiac muscle-rich miRNAs or myomiRNAs such as mir-208 (mir-208a, b) play crucial roles in CVD [85–87]. The miRNAs are natural, single-stranded, small RNA molecules that are not translated into proteins and yet serve the pivotal function of regulating gene expression. It is estimated that only 1% of the genomic transcripts in mammalian cells encode miRNA. Genes encoding for miRNAs are transcribed from DNA to produce a primary transcript (pri-miRNA). The pri-mRNA is then processed into a shorter precursor miRNA (pre-miRNA), which undergoes further processing to form a mature, single-stranded miRNA that is 18 to 24 nucleotides long. A mature miRNA binds to its mRNA target at their complementary sequences and downregulates gene expression by either inhibiting the mRNA translation to proteins or inducing mRNA degradation. Studies on experimental heart failure models have identified several miRNAs as differentially expressed (for a complete list of miRNAs that are differentially expressed in heart-failure models please see the review [88]). Thus a cumulative change in miRNA profile accompanies heart failure-associated cardiac pathology. In contrast, a detailed study by Naraba and Iwai has suggested that microRNA profiles of heart and kidney do not show significant changes in salt-sensitive hypertension [89]. These researchers constructed microRNA libraries using the kidneys of Dahl salt-sensitive and Lewis rats taking normal or high-salt diets (4 groups) and identified 91 previously reported and 12 new microRNAs and then compared their expression profiles in kidney and heart ventricles. They concluded that the microRNA system is unlikely to contribute to salt-sensitive hypertension in Dahl salt-sensitive rats. It has also been reported that plasma levels of some miRNAs (mir-1, mir-208, mir-133a, mir-423-5p, mir-499) can be used as biomarkers for myocardial injury [90–92]. The mir-143 has recently emerged as an obesity-induced miRNA that inhibits INS-stimulated Akt activation and impairs glucose metabolism [93]. 

**Figure 2:**
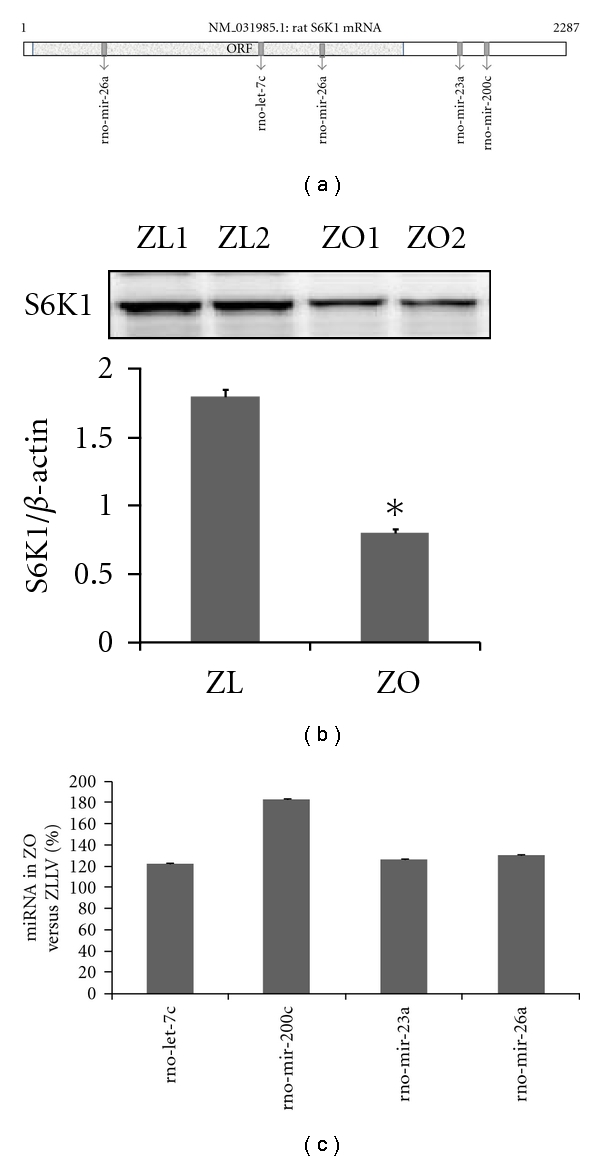
(a) Locations of miRNA binding sites on 2287bp S6K1 mRNA. Location of open reading frame (ORF) is marked. (b) Representative autoradiogram showing S6K1 protein levels in ZO and ZL LV tissues. *P < 0.05 for ZO versus ZL LV tissue. (c) % increase in miRNA levels in ZO LV tissues versus ZL LV tissues (*n* = 3 for each group, *P* for ZO versus ZL < 0.05).

Individual miRNAs can regulate several hundreds of genes and conversely a given gene can be regulated by multiple miRNAs. Knocking out the mTOR gene has shown to be embryonically lethal, however, knocking out S6K1 gene (RPS6KB1) seems to confer some beneficial health aspects to mice. Mice deficient in S6K1, though they have a small body size and reduced *β*-cell mass, are protected from INS resistance in conditions of overnutrition [94]. We observed that in ZO rat cardiac LV tissue, total S6K1 protein levels were significantly downregulated (Figure 2), and this prompted us to analyze whether or not a change in miRNA profile that modulates S6K1 translation and mRNA stability plays a role in reducing the protein levels of S6K1. To identify what miRNAs bind rat S6K1 mRNA, we performed a RegRNA analysis [95] of the 2287bp rat S6K1 mRNA. It was found that 298 putative miRNAs can bind rat S6K1 mRNA. We have initiated miRNA profiling studies of Zucker lean (ZL) and ZO cardiac tissues. The miRNA was isolated with mirVana mirNA isolation kit (Ambion Inc.) from fresh frozen tissues (*n* = 3 for each group), and was labeled with FlashTag Biotin HSR RNA Labeling Kit. Affymetrix miRNA GeneChip that carries 46,228 probes comprising 7,815 probe sets, including controls, was used for this study. The probes on this chip are derived from the Sanger miRBase miRNA database v11 (April 15, 2008, http://microrna.sanger.ac.uk) [96]. Data analysis was by miRNA QC tool and Significance Analysis of Microarrays (SAM) software (http://stat.stanford.edu/~tibs/SAM/). We compared the list of 298 miRNAs that can potentially modulate S6K1 expression with the list of statistically significant differentially expressed miRNAs in ZO LV tissue compared to ZL LV tissue. This analysis showed that only four of these S6K1-modulating miRNAs had a very modest, but statistically significant, increase in their expression compared to that of the ZL control cardiac tissues (Figure 2). 

**Figure 3:**
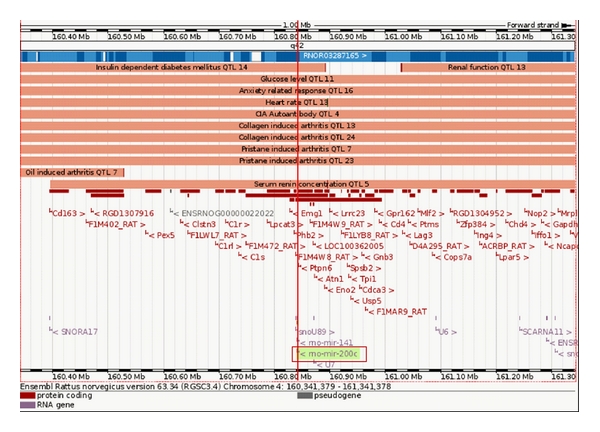
Location of rno-mir-200c on the chromosome 4 of rat with associated QTLs and protein coding regions as shown in Ensemble Rattus norvegicus version 63.34. rno-mir-200c is associated with Heart rate QTL 13.

Three of these miRNAs, rno-let-7c, rno-mir-23a, and rno-mir-26a, were among the abundantly expressed miRNAs in ZO cardiac tissue. This was not surprising since these miRNAs are shown to be expressed in heart and upregulated in experimental models for heart failure [88]. In contrast, rno-mir-200c was expressed modestly in ZO cardiac tissue. The rno-mir-200c is located on chromosome 4 and interestingly, QTLs associated with mir-200c include heart rate QTL (Figure 3). The mir-200c has emerged as a cell growth inhibitor and targets apoptosis inhibitor FAP-1 [97]. The mir-200c also regulates stem cell factors, and it has been proposed that targeting the ZEB1-miR-200 feedback loop can lead to a promising treatment for fatal tumors, such as pancreatic cancer [98]. Therefore, it is conceivable that the modest mir-200c upregulation in cardiac tissue from an overnutrition model reflects a compensatory mechanism to delicately balance mTORC1 signaling and to control hypertrophy. While none of the S6K1 targeting miRNAs showed robust increase in ZO cardiac tissue compared to that of ZL, it is possible that a modest upregulation of multiple miRNAs that target different regions of S6K1mRNA (Figure 2) may achieve optimum regulation of S6K1 expression (by either reducing translation or mRNA degradation) without completely ablating S6K1 and inhibiting its beneficial down-stream effects such as AT2R upregulation. 

## 4. A Possible Molecular Explanation for Exacerbation of Cardiomyopathy by Chronic Alcohol Consumption under Conditions of Overnutrition

Although light to moderate alcohol consumption is cardio-protective, regular heavy ethanol consumption results in a form of dilated cardiomyopathy characterized by reduced contractility, ventricular dilatation, cardiomyocyte apoptosis, and fibrosis, often progressing to cardiac failure [99–102]. The risk of alcoholic cardiomyopathy is greater in women than men for any given life time amount of alcohol [103]. 

The “2008-2013 Action Plan” by World health Organization estimates that noncommunicable diseases (NCDs) including CVDs, diabetes, cancers, and chronic respiratory diseases, constitute 60% of mortality globally and can be prevented by eliminating the shared risk factors that include alcohol abuse [104]. Epidemiological studies show either an inverted U-shape or a positive linear relationship between alcohol consumption and INS sensitivity [105]. Chronic heavy alcohol consumption has been associated with the development of INS-resistant syndrome [106, 107]. A recent study on patterns of alcoholic consumption and Metabolic Syndrome has reported that “drinking in excess of the dietary guidelines was associated with an increased risk of impaired fasting glucose/diabetes mellitus, hypertriglyceridemia, abdominal obesity, and high blood pressure” [108].

Chronic ethanol ingestion in rats resulted in decreased expression of GLUT4 accompanied by downregulation of INS receptor-beta subunit, INS receptor substrate-1 (IRS-1) in rat cardiac tissues [109]. Chronic ethanol feeding (12 weeks) to FVB mice resulted in glucose intolerance, impaired cardiac glucose uptake, cardiac hypertrophy, and contractile dysfunction [110]. Ethanol feeding had no effect on either the expression of INS receptor *β* and IRS-1 with or without INS stimulation in cardiomyocytes or basal phosphorylated INS receptor (Tyr1146), basal tyrosine, and Ser phosphorylated IRS-1 [110]. However, chronic alcohol ingestion significantly impaired INS-stimulated tyrosine phosphorylation of INS receptor, IRS-1, and Akt, S6K1. In alcohol dehydrogenase (ADH), transgenic mice ethanol-induced decrease in tyrosine phosphorylation of IRS-1 was further increased without affecting the INS receptor. Alcohol ingestion significantly enhanced INS stimulated Ser phosphorylation of IRS-1, the effect of which was exaggerated in ADH transgenic mice [110]. In contrast, mitochondrial aldehyde dehydrogenase-2 (ALDH2) overexpression attenuated alcohol-induced decrease in tyrosine phosphorylation of INS receptor and IRS-1. Moreover, increased Ser phosphorylation of IRS-1 and decreased phosphorylation of Akt caused by alcohol ingestion were reversed by ALDH2 overexpression [111]. These results favor the role of ethanol metabolism and acetaldehyde in alcohol-induced myocardial INS resistance and myocardial dysfunction. 

**Figure 4:**
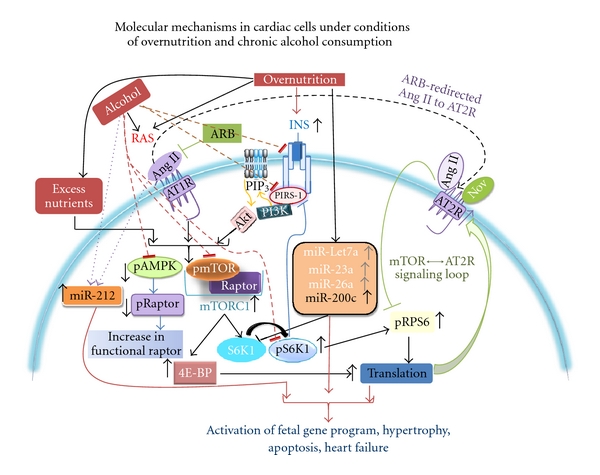
Established and putative signaling mechanisms activated by chronic alcohol consumption are superimposed on the signaling mechanisms activated by overnutrition to show how alcohol may exacerbate cardiac diseases in the setting of overnutrition. Dotted purple lines show alcohol mediated upregulation, and dotted orange lines show alcohol-induced inhibition of different signaling components.

Recent studies have shown that chronic alcohol treatment results in inhibition of mTORC1 signaling in cardiac tissues [59], and this effect of alcohol is involved in reduced protein synthesis and cardiac muscle waste. Inhibition of mTORC1 signaling by chronic alcohol consumption has also been reported in cerebral cortex [112]. Interestingly, this effect of alcohol was independent of TSC2 or Akt phosphorylation status suggesting that other mechanisms are involved in alcohol-mediated mTORC1 inhibition. The observation that alcohol inhibits mTORC1 signaling in the heart is particularly noteworthy since this effect of alcohol opposes overnutrition-mediated signaling in heart. As discussed in the previous sections, mTORC1 signaling plays a pivotal role in regulating cardiac health and pathology. Activation of mTORC1 signaling in heart tissue and cardiomyocytes underlies initiation of compensatory mechanisms such as upregulation of the AT2R that has cardio-protective effects. A delicate balancing of mTORC1 signaling in cardiac tissues under overnutrition conditions is mediated by mTOR*↔*AT2R signaling loop and a moderate downregulation of total S6K1 protein by changes in miRNA profiles. Therefore, it is conceivable that alcohol-mediated inhibition of mTORC1 signaling may attenuate natural compensatory mechanisms that help the heart to cope with overnutrition (Figure 4). 

Another effect of alcohol is its ability to downregulate mir-200a [113, 114] as shown by studies involving Lieber-DeCarli diet-induced alcoholic steatohepatitis mice models. The mir-200 family microRNAs are shown to function as inhibitors of growth in many cell types. Therefore, downregulation of mir-200 family microRNAs by alcohol can potentially increase growth and S6K1 protein levels. Alcohol-mediated upregulation of the micoRNA mir-212 is implicated in alcoholic liver disease [115]. Interestingly, mir-212 has emerged as an activator of fetal gene program [116, 117]. Chronic heart failure is characterized by LV remodeling and activation of the fetal gene program. The mir-212 is overexpressed in failing hearts. Additionally, transfection of isolated adult rat cardiomyocytes with a set of fetal miRNAs (miR-21, miR-129, and miR-212) induced cellular hypertrophy and activation of a fetal gene program [117]. Since alcohol is shown to up-regulate mir-212 in other cell types, it is conceivable that alcohol-mediated upregulation of mir-212 can be one of the mechanisms by which alcohol exacerbates cardiomyopathy. 

Activation of RAS may contribute to progression of alcoholic cardiomyopathy. Binge mode ethanol consumption in chronic alcohol abuse patients and heavy alcohol consumption is associated with increased plasma angiotensin levels [118]. Studies on alcohol ingestion and cardiac injury in dogs showed that activation of RAS was followed by a progressive fall in LV contractility during six months of alcohol ingestion [119]. Moreover, angiotensin receptor blocker irbesartan prevented these alcohol-induced decreases in LV and myocyte contraction [119]. Recent animal studies in which chronic alcohol consumption with superimposed binge mode of ethanol administration has been associated with upregulation of RAS in the heart [120]. However, ethanol ingestion alone usually does not result in severe cardiac injury seen in humans and robust activation of RAS [118]. In this regard, gene polymorphism for angiotensin converting enzyme (ACE) is associated with increased vulnerability to alcoholic cardiomyopathy [121]. 

One signaling cascade activated by ethanol and angiotensin is activation of mitogen-activated protein kinases (MAPKs) including ERK1/2. The activation of RAS and cardiac injury caused by chronic ethanol were associated with activation of ERK1/2 in cardiac tissues, and both activation of RAS and activation of ERK1/2 were reduced by inhibition of ERK1/2 signaling through administration of MEK inhibitor PD98059 [120]. In this regard, it is interesting to note that ethanol potentiates ERK1/2 activation induced by angiotensin in hepatocytes [122]. Ang II inhibited INS-induced glucose uptake in vascular smooth muscle cells in an ERK1/2-dependent manner, and increased Ser307 phosphorylation of IRS-1 was also inhibited by MEK inhibitor PD98059 [123]. Ang II causes INS resistance in cardiomyocytes which is sensitive to ERK1/2 inhibition [124]. In this regard, the role of mir-212 is noteworthy. Mir-212 has been shown to be upregulated by ethanol in intestinal cells [115] and Ang II in cardiac fibroblasts, which is associated with activation of ERK1/2 pathway [125]. Chronic ethanol ingestion also caused increased cardiac phosphorylation of CREB [126]. The significance of CREB phosphorylation emerges from a study reporting regulation of mir-212 in a CREB-dependent manner involving ERK1/2 activation [127]. Acetaldehyde causes activation of MAPKs including ERK1/2 in fetal human cardiac myocytes and ALDH2 transgene attenuated acetaldehyde-induced activation of phosphorylation of ERK1/2 and SAP/JNK [128]. Moreover, regulation of human microRNA-generating complex by MAPK/ERK pathway has also been reported [129]. These findings suggest possible interactions between alcohol and angiotensin on MAPK signaling and INS signaling through modulation of microRNA pathway in alcoholic cardiomyopathy.

Collectively, chronic alcohol consumption superimposed on overnutrition-mediated cardiac pathology can putatively induce the following signaling mechanisms: (1) inhibition of mTORC1 and attenuation of protective compensatory mechanisms; (2) inhibition of INS metabolic signaling via downregulation of INS receptor, IRS-1, GLUT4; (3) downregulation of growth inhibitory mir-200 family microRNAs and (4) upregulation of mir-212 (Figure 4).

It is suggested that mTOR inhibits autophagy in myocardium, and this inhibition of autophagy by mTOR can result in accumulation of abnormal proteins that lead to enhanced endoplasmic reticulum stress, apoptosis, and cardiac dysfunction [130, 131]. Inhibition of mTOR by Everolimus (Rapamycin) seems to accentuate autophagy and improve cardiac function [130–132]. In this context, a recent study that demonstrates alcohol-induced autophagy in myocardium [133] is particularly noteworthy. This study shows that chronic alcohol treatment-induced cardiac dysfunction is associated with attenuation of mTOR stimulatory phosphorylation and induction of autophagy in mice; however, ALDH2 overexpressing mice were protected from these effects. Interestingly, ALDH2-mediated protection of heart in this model of chronic alcoholism seems to be mediated through Akt-mTOR-STAT3 (Signal Transducer and Activator of Transcription 3) signaling [133]. These data further support the notion that mTOR activation is cardio-protective [133, 134]. They also highlight the need for a cautious approach towards treatments involving ablation of mTOR-signaling in heart since enhanced autophagy can also deteriorate heart condition. 

However, chronic alcohol intake also suppresses AMP kinase (AMPK) phosphorylation in myocardium [133]. Accumulating evidence suggests that AMPK is involved in GLUT4 translocation and glucose uptake [135–137]. Thus, inhibiting AMPK activation is an additional mechanism by which alcohol can dampen insulin metabolic signaling (Figure 4). Indeed, it is reported that in rats subjected to chronic alcohol consumption, the expression of AMPKalpha, and myocyte enhancer factor 2 (MEF2) is significantly reduced and this reduction is associated with GLUT4 decline [138].

## 5. Perspectives

In conditions of chronic alcohol intake and overnutrition, the potential compensatory mechanisms activated during overnutrition-related insulin resistance alone are overridden by alcohol-induced detrimental signaling (Figure 4). Moreover, since both overnutrition and alcohol activate RAS, and alcohol dampens insulin-signaling, insulin resistance is further exacerbated in cardiac tissue (Figure 4). In such conditions, upregulation of mir-212 by both alcohol and Ang II can lead to activation of fetal gene program and heart failure. Thus mir-212 can be a potential therapeutic target to protect the heart in conditions of overnutrition and chronic alcoholism. Recent advances in microRNA therapeutics are directed to develop effective strategies to block inappropriate expression of individual miRNAS that contribute to diseases [139–141]. In this context, the fact that miRNAs target multiple, functionally related genes (versus single genes), renders them powerful therapeutic tools. Antisense oligomers are known to work successfully in mammals [142–144]. A number of gene delivery systems have been developed to micromanage miRNAs by expressing such antisense oligonucleotides which include plasmids, and vectors based on adenoviruses, retroviruses, and lentiviruses. Cholesterol-conjugated antagomirs also provide an effective way to inhibit the activity of an miRNA. For example, the mir-21 antagomir/eraser is shown to be effective in alleviating cardiac fibrosis and hypertrophy since it derepresses the expression of mir-21target SPRY1 and increases myofibroblast apoptosis [144–146]. Therapeutic delivery of miRNAs can also be a very powerful strategy to regulate multiple genes at the same time. For example, therapeutic delivery of mir-200c is shown to ameliorate renal tubulointerstitial fibrosis [147]. However, a better understanding of the factors that regulate the rate and order of miRNA-mediated silencing of gene expression, colocalization of mRNA and miRNA, and miRNA turnover is crucial for optimizing the techniques for micromanipulating miRNAs as therapeutic targets.
